# Novel Small-Molecule Inhibitors of the SARS-CoV-2 Spike Protein Binding to Neuropilin 1

**DOI:** 10.3390/ph15020165

**Published:** 2022-01-28

**Authors:** Anja Kolarič, Marko Jukič, Urban Bren

**Affiliations:** 1 Faculty of Chemistry and Chemical Engineering, University of Maribor, Smetanova 17, SI-2000 Maribor, Slovenia; anja.kolaric2@um.si; 2 Faculty of Mathematics, Natural Sciences and Information Technologies, University of Primorska, Glagoljaška 8, SI-6000 Koper, Slovenia

**Keywords:** neuropilin 1, SARS-CoV-2, COVID-19, spike binding inhibitors, virtual screening, small-molecule antagonists, molecular docking, in vitro binding assay

## Abstract

Furin cleavage of the SARS-CoV-2 spike protein results in a polybasic terminal sequence termed the C-end rule (CendR), which is responsible for the binding to neuropilin 1 (NRP1), enhancing viral infectivity and entry into the cell. Here we report the identification of 20 small-molecule inhibitors that emerged from a virtual screening of nearly 950,000 drug-like compounds that bind with high probability to the CendR-binding pocket of NRP1. In a spike NRP1 binding assay, two of these compounds displayed a stronger inhibition of spike protein binding to NRP1 than the known NRP1 antagonist **EG00229**, for which the inhibition of the CendR peptide binding to NRP1 was also experimentally confirmed. These compounds present a good starting point for the design of small-molecule antagonists against the SARS-CoV-2 viral entry.

## 1. Introduction

Severe acute respiratory syndrome coronavirus 2 (SARS-CoV-2) is the causative agent of the coronavirus-induced disease 2019 (COVID-19) [1], which has spread worldwide. The pandemic resulted in a tremendous pressure on hospital care and caused the deaths of more than four million people [2]. While SARS-CoV-2 symptoms range from mild to severe, and in some cases fatal respiratory manifestations, its involvement in extrapulmonary indications has also been widely observed. Since it affects haematological, cardiovascular, renal, gastrointestinal, and hepatobiliary, endocrinological, neurological, ophthalmological, and dermatological systems [3], it is considered a multisystem disease for which successful treatments are necessary in the early stages of the infection in order to prevent more extensive health damage [4].

SARS-CoV-2 primarily utilises angiotensin-converting enzyme 2 (ACE-2) to mediate cell uptake [5], but alternative entry points have been identified, such as neuropilin-1 (NRP1) [6,7]. NRP1 was found to significantly enhance the infectivity of SARS-CoV-2 by increasing viral entry into host cells rather than strengthening viral binding [6]. Neuropilins (NRPs) represent a class of transmembrane glycoprotein receptors involved in various biological processes, including neuronal development and axon guidance, angiogenesis, vascular permeability, and immune functions [8,9]. They lack direct signalling capabilities and, therefore, act as co-receptors that bind a signalling molecule in addition to the primary receptor, thereby affecting ligand-receptor activity. NRP1 is implicated in several conditions of SARS-CoV-2 infection. The most pronounced is its association with the neurological manifestations of SARS-CoV-2, where it was discovered that NRP1 is elevated in the olfactory epithelial cells of infected humans, enhancing the entry of SARS-CoV-2 into the central nervous system [10]. The overexpression of NRP1 in olfactory cells also makes it a strong candidate for the mechanism of anosmia, which is very common in COVID-19 patients [11,12]. Moreover, it has also been suggested that NRP1 may be associated with additional neurological disorders such as headache, dizziness, hallucinations, or motor coordination disorders [10,13]. Furthermore, patients infected with SARS-CoV-2 are at a higher risk of increased blood clotting [14] and since NRP1 modulates blood clotting, it may also play a role in the pathology of blood clotting in COVID-19 patients [12]. Furthermore, NRP1 is involved in immune function, thus its role in the exaggerated immune response in severe COVID-19 cases has also been proposed [10,12]. The involvement of NRP1 in all listed functions makes it an ideal factor for the multisystemic effects of SARS-CoV-2 infection and, more importantly, a good target for attenuating viral infectivity and for preventing the associated disorders.

There exist two very similar isoforms, NRP1 and NRP2, which share 44% amino acid sequence identity and exhibit a similar structural domain organisation consisting of N-terminal extracellular domains, a single transmembrane domain, and a small intracellular C-terminal domain [15]. The individual extracellular regions are responsible for the binding of various endogenous ligands and trigger specific intercellular effects. The two CUB domains (a1/a2) bind semaphorins responsible for the functioning of the neural system, while the two factor V/VIII homology domains (b1/b2) bind vascular endothelial growth factor (VEGF) family ligands (mainly VEGF-A) involved in blood vessel development. The smallest MAM domain (c) mediates NRP multimerization [15,16]. A similar structural composition of NRP1 and NRP2 allows them to be involved in analogous biological processes yet to differ enough to provide selectivity for endogenous ligands [17]. Although NRP2 is an equally important target, only the involvement of NRP1 in COVID-19 disease has been studied and characterised in detail [10].

**Figure 1:**
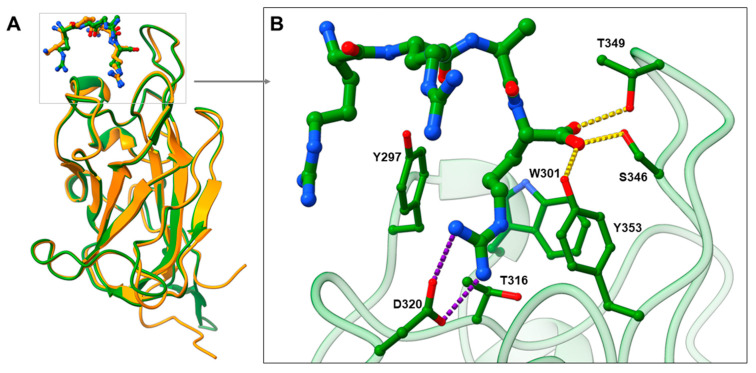
(**A**) Comparison of the binding modes of VEGF-A_164_ (in orange stick representation) and SARS-CoV-2 (in green stick representation) CendR motifs by superimposing VEGF-A-NRP1 (PDB ID: 4DEQ; [17] NRP1 in orange cartoon representation) and SARS-CoV-2-NRP1 (PDB ID: 7JJC; [6] NRP1 in green cartoon representation) complexes. (**B**) Zoomed view representing SARS-CoV-2 CendR binding mode into the b1 domain of NRP1, highlighting interactions with the key amino-acid residues (in green stick representation). Hydrogen bonding and salt bridge interactions are depicted as yellow and violet dashes, respectively.

SARS-CoV-2 entry into human cells is mediated by SARS-CoV-2 spike glycoproteins. The cleavage of the spike protein by host proteases is required for the binding to the host cell surface and cell entry. Accordingly, the spike is cleaved by the furin protease at the S1/S2 junction into the S1 subunit, which is responsible for the binding to host cell receptors, and the S2 subunit, which is responsible for fusion of the viral and cellular membranes [5,18]. After cleavage, the [R/K]XX[R/K] sequence called C-end rule (CendR) becomes exposed on the S1 subunit [18], which represents the key element for spike binding to NRP1. CendR is a common C-terminal sequence that allows the binding to the b1 domain of NRP1 in its highly conserved arginine binding pocket [19]. Endogenous VEGF family ligands use this CendR motif to activate signalling pathways [19], while viruses such as Epstein—Barr virus (EBV) [20] and human T-lymphotropic virus-1 (HTLV-1) [21] use CendR to promote host cell infection. Analogously, SARS-CoV-2 binds to the NRP1 b1 domain with its RRAR CendR motif to facilitate viral entry into the cell [6]. The suitability of the binding pocket to accommodate different ligands with the CendR terminals is evident from the comparison of the crystal complexes of VEGF-A_164_-NRP1 and SARS-CoV-2-NRP1 (Figure 1A), which show remarkable similarities in the binding mode of their CendR sequences. Amino acid residues Tyr297, Trp301, Thr316, Asp320, Ser346, Thr349, and Tyr353 were identified as critical for the SARS-CoV-2 binding and are virtually identical to the key interactions of VEGF-A_164_ with NRP1 [17]. The guanidine group of SARS-CoV-2 forms a salt bridge with Asp320, while its free carboxyl group interacts with Ser346, Thr349 and Tyr353 via hydrogen bonds. Tyr297 and Tyr353 are also interacting with the arginine side chains of CendR (Figure 1B) [6]. The binding of SARS-CoV-2 to NRP1 not only prevents the binding of VEGF-A, but also its signalling and function. Consequently, disruption of the VEGF-A/NRP1 complex by the SARS-CoV-2 spike protein was found to impair pain signalling and reduce neuropathic pain, which may contribute to the asymptomatic COVID-19 infections and further spread of the virus [22]. The spike protein of SARS-CoV-2 was associated with several amino-acid substitutions that affected its viral properties. However, the spike mutations do not appear to affect the binding of the CendR motif, as a high degree of conservation was observed for the Arg682 and Arg683 CendR residues, whereas the Ala684Val mutation appears to be consistently present. Since Ala684 does not form key interactions, its mutation appears to be without a significant effect [23]. Moreover, no spontaneous mutations were found for the CendR terminal Arg685 [24,25], which contributes most to the spike binding with the b1 domain of NRP1. Therefore, compounds that bind to the same amino-acid residues as CendR are likely to retain a high inhibition potential, even in the case of different variants of the SARS-CoV-2 virus. No information on the spontaneous mutations of the amino-acid residues in the CendR binding pocket of NRP1 could also be found.

**Figure 2:**
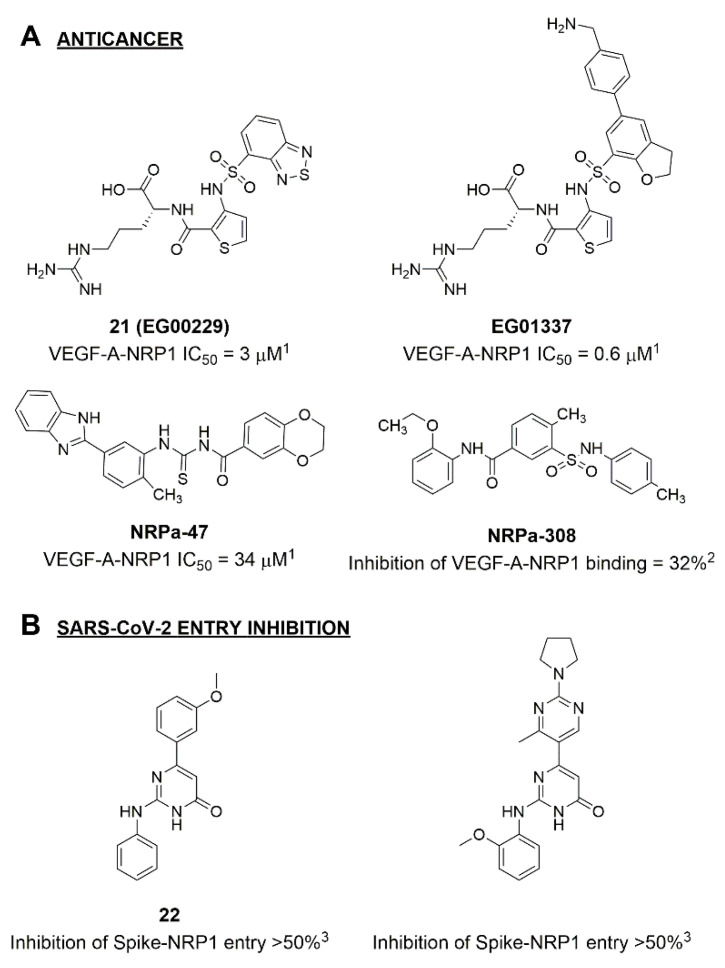
(**A**) Some of the best characterized antagonists of the VEGF-A binding to the NRP1 b1 domain. (**B**) Small-molecules identified as potential antagonists of the spike induced SARS-CoV-2 entry. ^1^ Cell-free bt-VEGF-A165 binding assay, measuring inhibition of VEGF binding to the b1 domain on NRP1 [35,38,39]. ^2^ Cell-free bt-VEGF-A165 binding assay measuring inhibition of VEGF binding to the b1 domain on NRP1 at a compound concentration of 10 μM [41]. ^3^ Vero-E6-TMPRSS2 cell-based assay measuring inhibition of SARS-CoV-2 spike protein dependent entry using GFP-expressing vesicular stomatitis virus (VSV) recombinant protein, encoding the SARS-CoV-2 spike protein [45].

Disrupting the key protein-protein interactions between NRP1 and VEGF-A at the b1 CendR binding site has long been the focus of drug discovery efforts, particularly for cancer therapy. As a result, several peptide [26,27,28,29,30,31,32,33,34], peptidomimetic [35,36,37,38], and small-molecule NRP1 antagonists (Figure 2A) have been developed that successfully interfere with the VEGF-A binding and prevent its further signaling as reported by Borriello et al. and Starzec et al. [39,40,41,42,43,44,45]. In peptide and peptidomimetic antagonists, the C-terminal arginine residue was generally retained to maintain interactions with the key amino acid Asp320 residue, whereas in small-molecule antagonists, this residue was replaced by a series of moieties. Interestingly, although the interaction with Asp320 was considered crucial for the binding, some studies suggested that a good affinity can also be achieved with antagonists lacking this interaction [40,42], expanding the possibilities for future structural modifications of small-molecule NRP1 antagonists. However, this interaction has still been recognised as critical for the SARS-CoV-2 binding [46], thus targeting it aims at successfully disrupting the SARS-CoV-2 NRP1 binding and is considered an attractive therapeutic approach to prevent viral entry. The well-known peptidomimetic NRP1 antagonist EG00229 (Figure 2A), which binds to the CendR-binding pocket and prevents the VEGF-A binding, was also evaluated for blocking the spike CendR binding in two different assays. In a binding assessment experiment, EG00229 inhibited the direct binding of the S1 CendR peptide to NRP1. Moreover, EG00229 decreased the SARS-CoV-2 infectivity in cells [6]. In the second experiment, EG00229 was tested in a spike-dependent assay for its ability to inhibit vesicular stomatitis virus using the SARS-CoV-2 spike protein for entry and fusion, but unfortunately it was found inactive. Nonetheless, two alternative compounds (Figure 2B) from this study displayed greater than 50% inhibition of spike-mediated viral entry into cells, representing the first step in the effort to develop small-molecule NRP1 antagonists for the treatment of SARS-CoV-2 infection [45].

With the aim of identifying non-peptide hit compounds capable of preventing spike CendR binding to the b1 domain of NRP1, we performed an extensive virtual screening campaign using molecular docking and a large library of small-molecules with drug-like properties. The compounds with the top scoring binding modes in the CendR binding pocket of NRP1 were further biologically evaluated for the spike binding blockade. We successfully identified two novel compounds with high inhibitory activity that exhibit a great potential for further developments of small-molecule antagonists of SARS-CoV-2 Sprot-NRP1 binding.

## 2. Results and Discussion

### 2.1. Molecular Docking and Selection of Top Compounds

**Figure 3:**
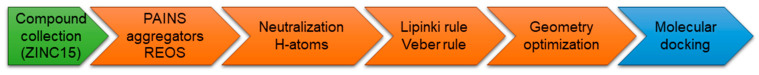
Library preparation for molecular docking calculations on the b1 domain of NRP1. The final library contained 956,355 molecules.

With the intention of identifying compounds that prevent the SARS-CoV-2 spike protein from binding to NRP1, a subset of the available compounds from the ZINC15 [47] library was prepared and screened against the CendR binding site on the NRP1 b1 domain. Library preparation (Figure 3) was performed in order to filter out structural faults, eliminate known and predicted aggregators, allow elements: H, C, N, O, F, S, Cl, Br, I, P, filter for pains (pan-assay interference compounds) [48,49] and REOS (rapid elimination of swill) structures to eliminate reactive and labile functional groups as well as to apply Lipinski [50] and Veber [51] medicinal chemistry filters. Therefore, KNIME software with RDKit software nodes was used to compare all structures in the library to the selection of SMARTS-formatted flags and to remove hits from the database [52].

Two screenings were performed, the first with lower search efficiency to estimate the binding of the compounds and to select the compounds with the best predicted binding poses as well as the second screening with a higher search efficiency. We employed the GOLD implementation of ChemPLP empirical scoring function in order to achieve the best HTVS results [53]. Finally, the 20 highest scoring compounds predicted to have hydrogen bonding interactions with Asp320, Ser346, Thr349 and Tyr353 were selected for the subsequent biological evaluation (Table 1). Chemical descriptor analyses of the docking-hits also confirmed the suitable calibration of input libraries as hit compounds covered the complete and similar chemical space.

### 2.2. In Vitro Evaluation of the Spike Binding Inhibition to NRP1

The ability of the compounds to inhibit the binding of the spike S1 protein to the b1 domain of NRP1 was evaluated by an in vitro spike-NRP1 binding assay, in which the potential antagonist competes with the spike S1 protein for the binding to NRP1. The results presented in Table 1 show that all docked compounds were able to inhibit the binding of the spike S1 to a certain extent, but only compounds **16** and **17** were able to prevent more than 60% of the binding. To evaluate the results, the inhibitory activities of the tested compounds were compared with the inhibitory activity of EG00229 (**21**) [35] and compound **22** [45], which had previously shown inhibition of spike-mediated cell entry, and which were used as controls in our assay. Compound **21** displayed 50.57 ± 2.26% inhibition of spike S1 binding to NRP1, while **22** was less active with 28.71 ± 0.80 inhibition. According to our results, the compounds **16** and **17** act as stronger antagonists of spike binding than compound **21**, suggesting that they represent a good starting point for further development of small-molecule SARS-CoV-2 antagonists.

### 2.3. Prediction of Compounds’ ***16*** and ***17*** Binding to NRP1

**Figure 4:**
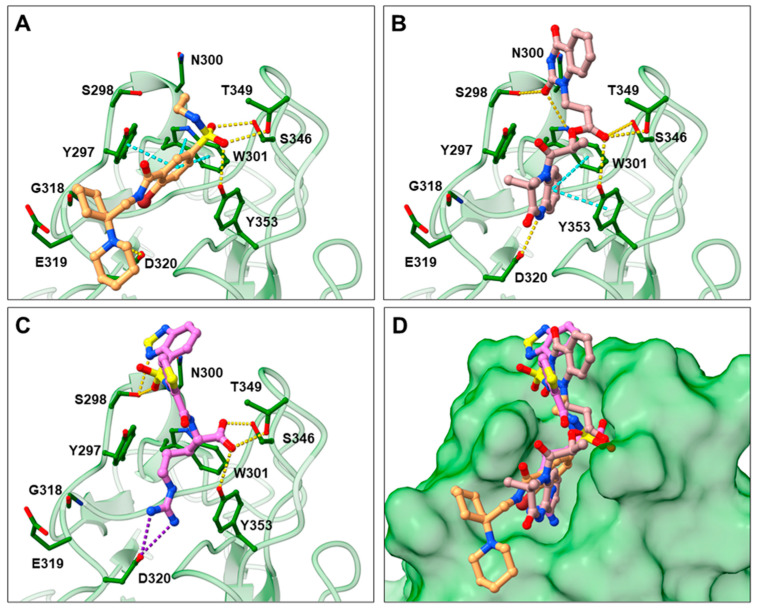
Predicted binding poses of compounds **16** (**A**) and **17** (**B**) as well as the binding mode of compound **21** ((**C**); PDB ID 3i97) [35] within the SARS-CoV-2 CendR binding pocket on the b1 NRP1 domain (PDB ID: 6FMC) [38]. NRP1 is depicted in green cartoon representation, while ligands and key amino acids are in stick representation. Hydrogen, salt bridge and π-π stacking interactions are depicted as yellow, violet, and cyan dashes, respectively. Overlay of the predicted binding modes of compounds **16** and **17** with the binding pose of compound **21** (**D**).

In the spike protein NRP1 binding assay, compounds **16** and **17** were identified as the most active, so we examined their predicted binding modes in more detail. As indicated with the crucial criteria for selecting the top 20 compounds, both **16** and **17** predictably form interactions with key amino acids Asp320, Ser346, Thr349, and Tyr353. The predicted binding pose of compound **16** is shown in Figure 4A, where the sulfonyl group forms hydrogen bonds with Ser346, Thr349 and Tyr353, while the amino group on the cyclohexane ring forms a salt bridge interaction with Asp320. The single aromatic ring is involved in π-π stacking with Tyr297 and Trp301. The predicted binding mode of compound **17** in Figure 4B shows that the carbonyl group of the ester forms hydrogen bonds with the key amino acids Ser346, Thr349 and Tyr353. Moreover, the free amino group of the dihydroquinoxalinone moiety is involved in hydrogen bonding with Asp320. The same moiety also participates in π-π stacking with Trp301 and Tyr353. Furthermore, the compound is bound to Ser298 and Trp301 via two different carbonyl groups of the dihydroquinoxalinedione moiety. Comparing the predicted binding modes of compounds **16** and **17** with the binding pose of compound **21** (Figure 4C, PDB ID: 3i97) [35], the binding mode of **17** is consistent with the conformation of compound **21**. While the functional groups of **17** and **21** extend towards the upper polar region of the receptor formed by amino-acid residues Ser298 and Asn300, thus enhancing the binding, compound **16** extends towards, but does not interact with, the lower open region of the binding pocket surrounded by amino-acid residues Gly318 and Glu319 (Figure 4D). Therefore, targeting these residues could further stabilize the binding of the ligand in the lower part of the binding pocket. Moreover, compound **16** has the potential to introduce functional groups that occupy the upper part of the binding pocket. The importance of amino acid residues in the upper (Ser298 and Asn300) and lower (Gly318 and Glu319) parts of the binding pocket has been previously considered as a possibility for the design of compounds that could fully occupy the binding pocket and thus enhance the binding, potentially leading to stronger antagonistic effects [45].

## 3. Materials and Methods

### 3.1. Molecular Docking Calculations

A subset of “now” available compounds was downloaded from the ZINC15 database [47]. The library was prepared for screening using an in-house derived KNIME [54] protocol (Figure 3) removing PAINS [48,49], known aggregators [55] and REOS structures to eliminate reactive functional groups as well as providing neutralization, drug-like properties, adding hydrogens and optimizing geometry with RDKit software nods, which yielded ∼950K compounds.

The structure of the NRP1 b1 domain with the highest available resolution of 0.90 Å (PDB ID: 6FMC) [38] was selected for subsequent docking calculations. Prior to molecular docking studies, the protein was prepared using the Prepare Protein Wizard in the Maestro 12.6 program (Release 2020–4, Schrödinger, LLC, New York, NY, USA). The high throughput virtual screening of the library was performed using the GOLD (Version 2020.2.0, CCDC, Cambridge, UK) docking suite within the CendR binding pocket of the NRP1 b1 domain [56]. The experimental coordinates of the co-crystallized ligand (EG01377) were used to define the binding site (cavity radius of 17 Å). The molecular docking protocol was validated by triple docking of the co-crystallized ligand (EG01377) to reproduce its spatial conformation and orientation (Supplementary Materials: Figure S1). The root-mean-square deviation (RMSD ≤ 2.0 Å) between each calculated docking pose and the co-crystallized ligand conformation served as the crucial criterion for the quality of all structure-based settings [57]. Furthermore, we calculated the receiver operating characteristic (ROC) curve to validate the performance of classifier docking method. We selected a set of known NRP1 inhibitors from the ChEMBL database (CHEMBL5174) with experimental values consisting of compounds with reported IC_50_ (28 compounds) as well as of compounds with reported K_d_ employing the cutoff of 5 µM (27 compounds), following this we concatenated and prepared the ligands with Schrödinger SMD suite. We then created a testing database by the addition of the negative control compounds that were calculated decoys based on employed actives using DUD-E: A Database of Useful (Docking) Decoys [58]. Upon using 3% and 10% of actives in the test database, we obtained a ROC AUC of 0.76 and 0.74, respectively, indicating that the docking protocol can indeed identify active compounds and produce enriched libraries. Identical settings and technical parameters of the GOLD genetic algorithm (automatic) were used for all calculations (Supplementary Materials: GOLD configuration file). Hydrogen bonding constraints were set for residues Asp320, Ser346, Thr349, and Tyr353 to favour the formation of specific hydrogen bonds. The calculated docking poses were evaluated according to two criteria. First, for each docking pose, the distance between the H-bond donor/acceptor atoms of the ligand and the H-bond donor/acceptor atoms of the key amino acids (Asp320, Ser346, Thr349, and Tyr353) were measured. Here, the binding poses with a distance from each key donor or acceptor atom greater than 2.5 Å were filtered out by an internally developed application. Among the binding poses meeting these hydrogen-bonding criteria, poses with a ChemPLP greater than 65 were considered acceptable. Compounds belonging to these poses (∼700 compounds) were subsequently subjected to more accurate docking calculations using analogous settings with 200% search efficiency. Finally, the predicted binding poses were ranked based on the ChemPLP scoring (Supplementary Materials: Table S1), and the highly ranked poses were also visually scored using the Maestro 12.6 program (Release 2020–4, Schrödinger, LLC, New York, NY, USA). Among the 20 compounds predicted to have hydrogen bonding interactions with Asp320, Ser346, Thr349, and Tyr353, all 20 were selected for biological evaluation.

### 3.2. Inhibition of SARS-CoV-2 Spike Binding to NRP1

The selected compounds were obtained from MolPort Inc. (Riga, Latvia; Supplementary Materials: Table S1). The COVID-19 Spike-NRP1 Binding Assay Kit was purchased from RayBiotech Life, Inc. (Peachtree Corners, GA, USA) to measure the binding affinity of spike S1 to NRP1 in the presence of a potential antagonist. The assay was performed on 96-well plates coated with recombinant NRP1 according to the manufacturer’s protocol. Potential antagonists in DMSO (Sigma-Aldrich, St. Louis, MO, USA) and the recombinant spike S1 protein were diluted with assay diluent and 100 μL were added to the wells. Since compound **21** was in the form of a trifluoroacetic acid salt, we converted it to the neutral form by adding one molar equivalent of triethylamine (Merck, Kenilworth, NJ, USA) to the solution of the compound, spike, and assay buffer. The plate was incubated at room temperature for 2.5 h using gentle shaking. Unbound S1 was then removed with a wash solution, and 100 μL of a mouse anti-S1 IgG detection antibody were added and incubated at room temperature for 1 h using gentle shaking to establish binding to the S1-NRP1 complex. After washing, 100 μL of an HRP-conjugated secondary anti-mouse IgG were added to the wells and incubated for 1 h with gentle shaking. Finally, after washing, 100 μL of 3,3′,5,5′-tetramethylbenzidine (TMB) substrate were added to the wells and incubated for an additional 30 min using gentle shaking. In this step, the horseradish peroxidase (HRP) reacted with the TMB solution to produce a blue colour proportional to the amount of S1 bound. The HRP-TMB reaction was quenched by the addition of 50 μL of the stop solution, resulting in a colour change from blue to yellow. The intensity of the yellow colour was then measured at 450 nm. The positive control (spike S1), vehicle control (DMSO), triethylamine control, and potential antagonist samples were all run in triplicate. The percentage of binding inhibition of spike S1 was determined using the following equation: BI = [1 − (potential antagonist sample − vehicle control)/positive control] × 100. The screening was performed at a potential antagonist concentration of 100 μM. The VEGF-A antagonist EG00229 (**21**) [35] and compound **22** [45], which possesses the ability to inhibit SARS-CoV-2 mediated entry, were used as controls at the identical concentration of 100 μM.

## 4. Conclusions

In the present work, we identified novel small-molecule inhibitors of spike S1 protein binding to NRP1 that could weaken SARS-CoV-2 infectivity. Compounds **16** and **17** displayed more than 60% inhibition and were more active than the EG00229 control (compound **21**), a known antagonist that prevents VEGF-A binding to NRP1 and has also been shown to block spike protein binding to NRP1. These results represent starting hit compounds suitable for additional in vitro and cell-based biological evaluations, with the ultimate goal of discovering and optimizing small-molecule inhibitors of SARS-CoV-2 spike-NRP1 binding with in vivo activity. As the COVID-19 pandemic seems to be far from over, novel medicinal chemistry approaches and the design of new drugs or biochemical probes are urgently needed.

## Figures and Tables

**Table 1:** Inhibition of the spike S1 binding to NRP1 by the compounds selected using molecular docking calculations.

Compound	Structure	ChemPLP Score ^1^	Inhibition of Spike-NRP1 Binding [%] ^2^
**1**	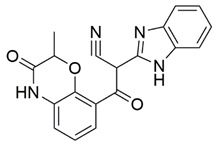	75.5	45.43 ± 1.58
**2**	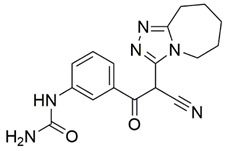	74.5	34.81 ± 1.09
**3**	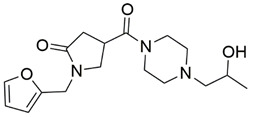	74.0	16.35 ± 1.95
**4**	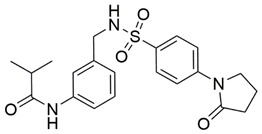	76.0	25.38 ± 0.82
**5**	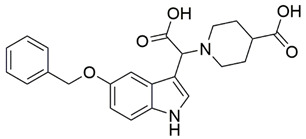	75.4	15.67 ± 1.02
**6**	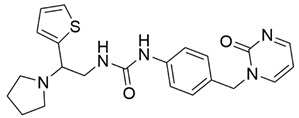	75.0	35.62 ± 1.43
**7**	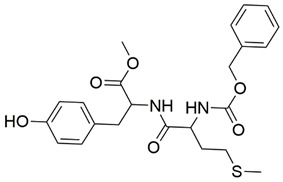	77.3	9.37 ± 0.59
**8**	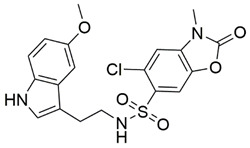	77.1	13.00 ± 6.39
**9**	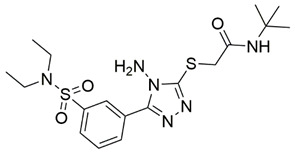	75.7	35.25 ± 4.35
**10**	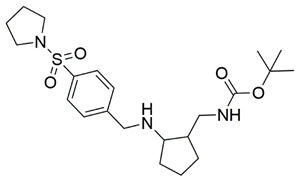	73.1	41.52 ± 0.57
**11**	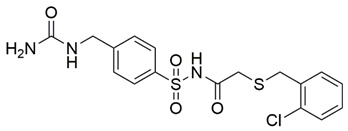	73.8	32.16 ± 1.28
**12**	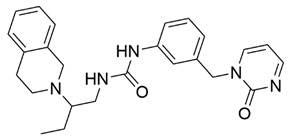	80.7	38.71 ± 1.68
**13**	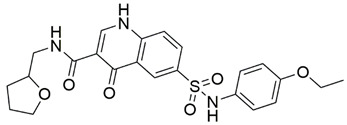	74.3	31.86 ± 2.79
**14**	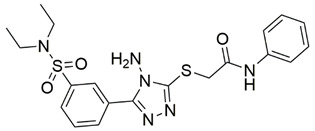	77.3	19.46 ± 2.37
**15**	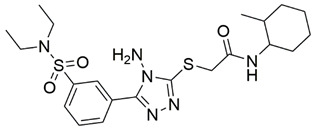	76.7	25.48 ± 5.11
**16**	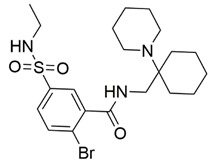	74.6	61.44 ± 2.48
**17**	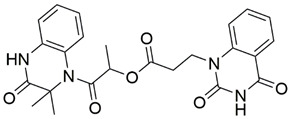	77.1	63.58 ± 1.27
**18**	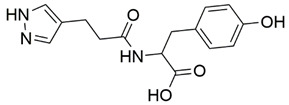	73.0	16.88 ± 0.31
**19**	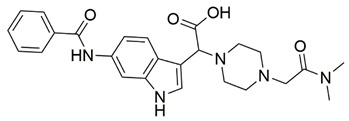	72.7	9.73 ± 0.04
**20**	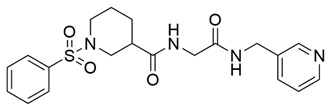	72.1	17.56 ± 1.65
**21** **EG00229** [33]	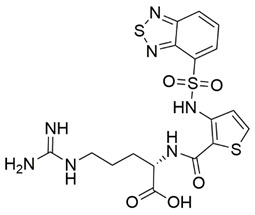		50.57 ± 2.26
**22** [43]	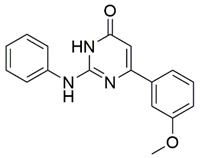		28.71 ± 0.80

^1^ Molecular docking score. ^2^ Inhibition of the spike S1 binding to NRP1 obtained at the compound concentration of 100 μM and presented as mean ± SD percentage of three measurements.

## Data Availability

Data sharing not applicable.

## Supplementary Materials

The following are available online at https://www.mdpi.com/article/10.3390/ph15020165/s1, Figure S1: Molecular docking validation, GOLD configurational file, Table S1: ZINC15 names and MolPort IDs of selected compounds.
