# Supplementary material for: Novel Small-Molecule Inhibitors of the SARS-CoV-2 Spike Protein Binding to Neuropilin 1
Source: Pharmaceuticals (Basel). 2022 Jan 28;15(2):165. doi: 10.3390/ph15020165 (PMC8879887; doi:10.3390/ph15020165)
Supplement: Supplementary file 1 [file pharmaceuticals-15-00165-s001.zip › pharmaceuticals-1463697-supplementary.pdf]

Supplementary Materials

Novel Small-Molecule Inhibitors of the SARS-CoV-2 Spike Protein Binding to Neuropilin 1

Anja Kolarič [1], Marko Jukič [1,2,*] and Urban Bren [1,2,*]

[1] Faculty of Chemistry and Chemical Engineering, University of Maribor, Smetanova 17, SI-2000 Maribor, Slovenia

[2] Faculty of Mathematics, Natural Sciences and Information Technologies, University of Primorska, Glagoljaška 8, SI-6000 Koper, Slovenia

[*] Correspondence: marko.jukic@um.si; Tel: +386 2 2294 428 (M.J.) and urban.bren@um.si; Tel: + 386 2 22 94 421 (U.B.)

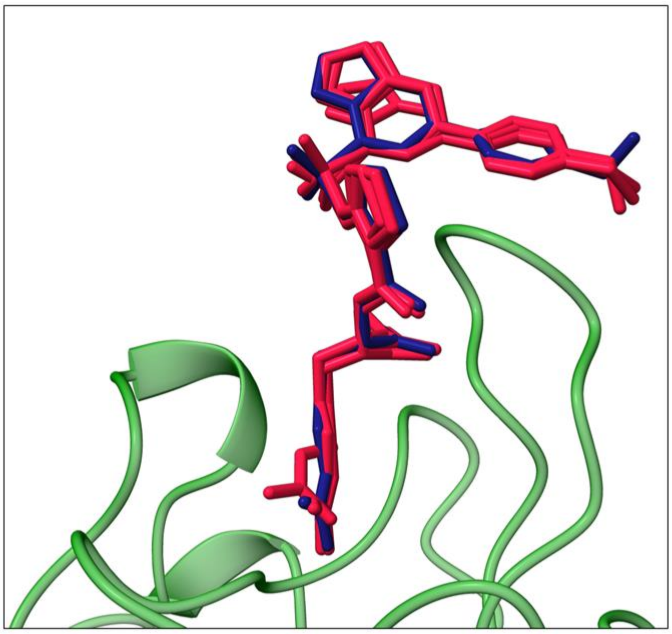

Figure S1. Spatial comparison of the natively present EG01377 conformation and its calculated docked poses for the molecular docking validation. The co-crystallized conformation is represented in blue, while its re-produced docked poses are depicted in red. NRP1 is in colored in green.

GOLD configuration file

AUTOMATIC SETTINGS
autoscale = 2

POPULATION
popsiz = auto
select_pressure = auto
n_islands = auto
maxops = auto
niche_siz = auto

GENETIC OPERATORS
pt_crosswt = auto
allele_mutatewt = auto
migratewt = auto

FLOOD FILL
radius = 10
origin = 0 0 0
do_cavity = 1
floodfill_atom_no = 0
floodfill_center = cavity_from_ligand 17 atoms

DATA FILES
param_file = DEFAULT
set_ligand_atom_types = 1
set_protein_atom_types = 0
tordist_file = DEFAULT
make_subdirs = 0
save_lone_pairs = 0
fit_points_file = fit_pts.mol2
read_fitpts = 0

FLAGS
internal_ligand_h_bonds = 0
flip_free_corners = 0
match_ring_templates = 0
flip_amide_bonds = 0
flip_planar_n = 1 flip_ring_NRR
flip_ring_NHR
flip_pyramidal_n = 0
rotate_carboxylic_oh = flip
use_tordist = 1
postprocess_bonds = 1
rotatable_bond_override_file = DEFAULT
solvate_all = 1

TERMINATION
early_termination = 1
n_top_solutions = 3
rms_tolerance = 1

CONSTRAINTS
force_constraints = 0

COVALENT BONDING
covalent = 0

SAVE OPTIONS
save_score_in_file = 1
save_protein_torsions = 0
clean_up_option save_top_n_solutions 3
clean_up_option
delete_redundant_log_files
clean_up_option
delete_all_initialised_ligands
clean_up_option delete_empty_directories
clean_up_option delete_rank_file
clean_up_option delete_all_log_files
output_file_format = MACCS

FITNESS FUNCTION SETTINGS
initial_virtual_pt_match_max = 3
relative_ligand_energy = 1
gold_fitfunc_path = plp
score_param_file = DEFAULT

CONSTRAINTS
constraint protein_h_bond 10.0000
0.005000 1215 1305 1167 411 754 755

Table S1. ZINC15 [1] name and MolPort ID (vendor) of top 20 compounds obtained from molecular docking.

COMPOUND	ZINC15 NAME	MolPort ID
**1**	ZINC000390834636	MolPort-039-314-631
**2**	ZINC000065529168	MolPort-019-672-384
**3**	ZINC000070040863	MolPort-020-084-791
**4**	ZINC000024451999	MolPort-009-171-237
**5**	ZINC000020732290	MolPort-006-814-244
**6**	ZINC000057997476	MolPort-009-073-388
**7**	ZINC000015721546	MolPort-002-516-537
**8**	ZINC000012428013	MolPort-002-749-663
**9**	ZINC000003295631	MolPort-004-259-418
**10**	ZINC000095995534	MolPort-028-795-075
**11**	ZINC000058026820	MolPort-009-102-148
**12**	ZINC000048326459	MolPort-009-073-421
**13**	ZINC000008584635	MolPort-007-627-531
**14**	ZINC000003271207	MolPort-004-257-684
**15**	ZINC000003319242	MolPort-004-262-387
**16**	ZINC000008708441	MolPort-005-566-437
**17**	ZINC000012769255	MolPort-005-749-385
**18**	ZINC000426477237	MolPort-042-631-013
**19**	ZINC000036360945	MolPort-008-317-429
**20**	ZINC000009422900	MolPort-005-913-364
**21** (EG00229) [2]		MolPort-046-416-868
**22** [3]		MolPort-003-000-781

REFERENCES

1. ZINC. Available online: https://zinc15.docking.org/ (accessed Oct 6, 2021)
2. Jarvis, A.; Allerston, C. K.; Jia, H.; Herzog, B.; Garza-Garcia, A.; Winfield, N.; Ellard, K.; Aqil, R.; Lynch, R.; Chapman, C.; Hartzoulakis, B.; Nally, J.; Stewart, M.; Cheng, L.; Menon, M.; Tickner, M.; Djordjevic, S.; Driscoll, P. C.; Zachary, I.; Selwood, D. L. Small molecule inhibitors of the neuropilin-1 vascular endothelial growth factor A (VEGF-A) interaction. J. Med. Chem. 2010, 53, 2215-2226. DOI: 10.1021/jm901755g.
3. Perez-Miller, S.; Patek, M.; Moutal, A.; Duran, P.; Cabel, C. R.; Thorne, C. A.; Campos, S. K.; Khanna, R. Novel compounds targeting neuropilin receptor 1 with potential to interfere with SARS-CoV-2 virus entry. ACS Chem. Neurosci. 2021, 12, 1299-1312. DOI: 10.1021/acschemneuro.0c00619.
